# Fat Graft as Regenerative Treatment of Facial Manifestations of Systemic Sclerosis: A Systematic Review on the Role of Adipose Tissue‐Derived Stem Cells and on Surgical Outcomes to Define a New Standardised Injection Protocol

**DOI:** 10.1111/wrr.70045

**Published:** 2025-05-19

**Authors:** A. Arena, S. Troise, F. De Francesco, M. Apolito, U. Committeri, G. Salzano, A. Romano, P. Bonavolontà, V. Abbate, R. Nocini, G. Dell’Aversana Orabona

**Affiliations:** ^1^ Maxillofacial Surgery Unit, Department of Neurosciences Reproductive and Odontostomatological Sciences, University Federico II of Naples Naples Italy; ^2^ Department of Reconstructive Surgery and Hand Surgery AOU ‘Ospedali Riuniti Ancona Italy; ^3^ Accademia del Lipofilling, Research and Training Center in Regenerative Surgery Jesi Italy; ^4^ Maxillofacial Surgery Unit, University Hospital of Terni Terni Italy; ^5^ Department of Otorhinolaryngology University Hospital of Verona Verona Italy

**Keywords:** adipose tissue‐derived stem cells, facial scleroderma, fat grafting, injection protocol, systemic sclerosis

## Abstract

Facial symptoms of systemic sclerosis (SSc)—such as reduced skin elasticity, fibrosis and microstomy—significantly impact quality of life. In recent years, autologous fat grafting has emerged as a promising treatment for these issues, but determining the optimal timing and techniques for fat injection remains a challenge for surgeons. Our study aimed to perform a systematic review of the available literature to establish a standardised protocol for this procedure. We reviewed all relevant studies published up to 18 August 2023, focusing specifically on diffuse facial scleroderma. In addition to clinical reports, we included articles discussing the pathophysiological mechanisms behind the effects of adipose stem cells. A total of 18 articles were analysed, revealing a range of methods and timelines for the procedure. The volume of fat injected varied from 6 cc for perioral treatment to 72 cc for a full‐face approach, with treatment intervals ranging from one session per year to one every 3 months. On average, around 50% of the fat was reabsorbed within 6 months. Adipose stem cells were identified as a key factor in both tissue regeneration and fat resorption rates. This review supports the effectiveness of autologous fat grafting for facial scleroderma, emphasising the role of adipose stem cells. For optimal results, two procedures spaced 3–6 months apart, followed by annual maintenance, are recommended. Consistent fat volumes in different facial areas are essential to achieve longer‐lasting outcomes and minimise resorption.

## 1 Introduction

Systemic sclerosis (SSc), also called scleroderma, is a rare, immune‐mediated rheumatic disease characterised by fibrosis of skin and internal organs and by vasculopathies. The manifestations of this disease are very variable and can drastically reduce the quality of life or can be potentially fatal [1]. In fact, morbidity and mortality are higher than any other rheumatic pathologies, except for patients with only diffuse cutaneous sclerosis who have a better survival.

Although SSc is multisystemic, skin involvement remains challenging and is often used to assess the prognosis of the disease. The progression of cutaneous SSc goes through three stages: edematous, sclerotic and atrophic. In the advanced form, the skin manifestations are characterised by loss of elasticity and fibrosis. These cutaneous alterations cause the typical ‘mask appearance’ to the face with microstomy and reduces buccal opening, that compromise the functional and aesthetic features of the face. The typical first‐line therapy for patients with early progressive cutaneous disease is Methotrexate. Rituximab is a viable alternative in case of diffuse skin form with joint involvement, and it is the first choice in case of cutaneous manifestations associated with interstitial lung disease [2]. Microstomy can have benefits with botulinum toxin treatment and hyaluronic acid injection [3].

Recently, the adipose tissue grafting has been recognised as a viable treatment option that can improve and slow down the skin manifestations of SSc, thanks to the pro‐angiogenic and immunosuppressive effect and the differentiation potential of the adipose‐derived stem cells (ASCs) [4]. In particular, ASCs seem to reduce collagen deposition in various in vitro and in vivo fibrosis models, encompassing pulmonary fibrosis, hypertrophic scar fibrosis and radiation‐induced fibrosis [5]. As reported in the literature, however, there are still debated aspects regarding the use of fat grafting. First of all, the choice of the correct phase to start the treatment is crucial to prevent the disease from being aggravated by the administration of the fat graft. According to the literature, the post‐treatment fat retention at 6 months after the procedure is only 50%. Therefore, an over‐injection of about 20% is advisable, considering at the same time that an excessive injection can cause increased skin tension or, in the most serious cases, necrosis and that increased fat grafting can also decrease fat graft take [5, 6].

As described, there is no common line on the number of ideal treatments to perform, on the interval time between one injection and the next, and on the correct follow‐up to evaluate the benefits of the treatment.

Therefore, a systematic review of the literature was conducted on fat grafting treatment in patients with facial manifestations of SSc, considering the doubts that currently exist on this topic. The aim of this study was to focus on the main aspects found in the literature about methods and timing of the procedure, clarifying current uncertainties and the role of ASCs to define a new injection protocol.

## 2 Materials and Methods

### 2.1 Study Protocol

The review was performed according to the protocol of the Preferred Reporting Item for Systematic Reviews and Meta‐analyses (PRISMA) statement [7]. The review protocol was designed a priori, defining methods for collecting, extracting and analysing the data. Two authors independently evaluated data from eligible studies, which were then checked by a third author.

### 2.2 Search Strategy and Eligibility Criteria

The literature research was conducted using different databases: PubMed, Web of Sciences, MEDLINE, Embase, Scopus, Google Scholar and Cochrane Library. The search was performed using the combination of the following keywords: ‘scleroderma’ OR ‘systemic sclerosis’ AND ‘fat graft’ OR ‘Lipofilling’ OR ‘fat transfer’ OR ‘fat transplant’ OR ‘adipose tissue‐derived stem cells’. The research covered all the literature on the topic up to the 18 August 2023. Duplicates were selected and then removed using Endnote online software (Clarivate Analytics). Only the papers in English language were included in the study; similarly, only the studies on humans and the articles with an abstract were considered for the review. The records assessed for the eligibility were fully read and screened.

In the present systematic review, all the studies that evaluated the efficacy of autologous fat transfer for the treatment of the facial manifestations of SSc were included; the papers had to contain information about the surgical procedure, the amount of injected fat, timing of procedures and outcomes. Only the studies that had as primary endpoint the description of the injection methods and the obtained outcomes, and the molecular basis of fat transplantation were considered for the review. The articles that considered other causes of facial sclerosis or localised form of scleroderma, such as ‘coup de sabre’ and morphea, were excluded. Similarly, the papers about other types of treatment or about the combination of fat with plasma rich in platelets (PRP) or hyaluronic acid were excluded. Moreover, articles explaining the pathophysiological mechanism of action of human ASCs on facial scleroderma were equally considered and included in the review.

### 2.3 Data Extraction

Data extracted from the included studies were about: the type of study, the number of analysed cases, the type of used surgical technique, the timing of the performed procedures, the obtained outcomes and the complications that occurred. In particular, the review analysed: ‐ the processing mode of adipose tissue, such as centrifugation, sedimentation or filtration;‐the amount of fat injected into specific facial regions;‐how many procedures were performed in 1 year and how many months apart; the outcomes in terms of improvement of skin elasticity, mouth opening, aesthetic appearance and pain;‐the complications of the procedure;‐the pathophysiological mechanism of action of adipose tissue and, in particular, of the stem cells.

## 3 Results

### 3.1 Studies Selection

**FIGURE 1:**
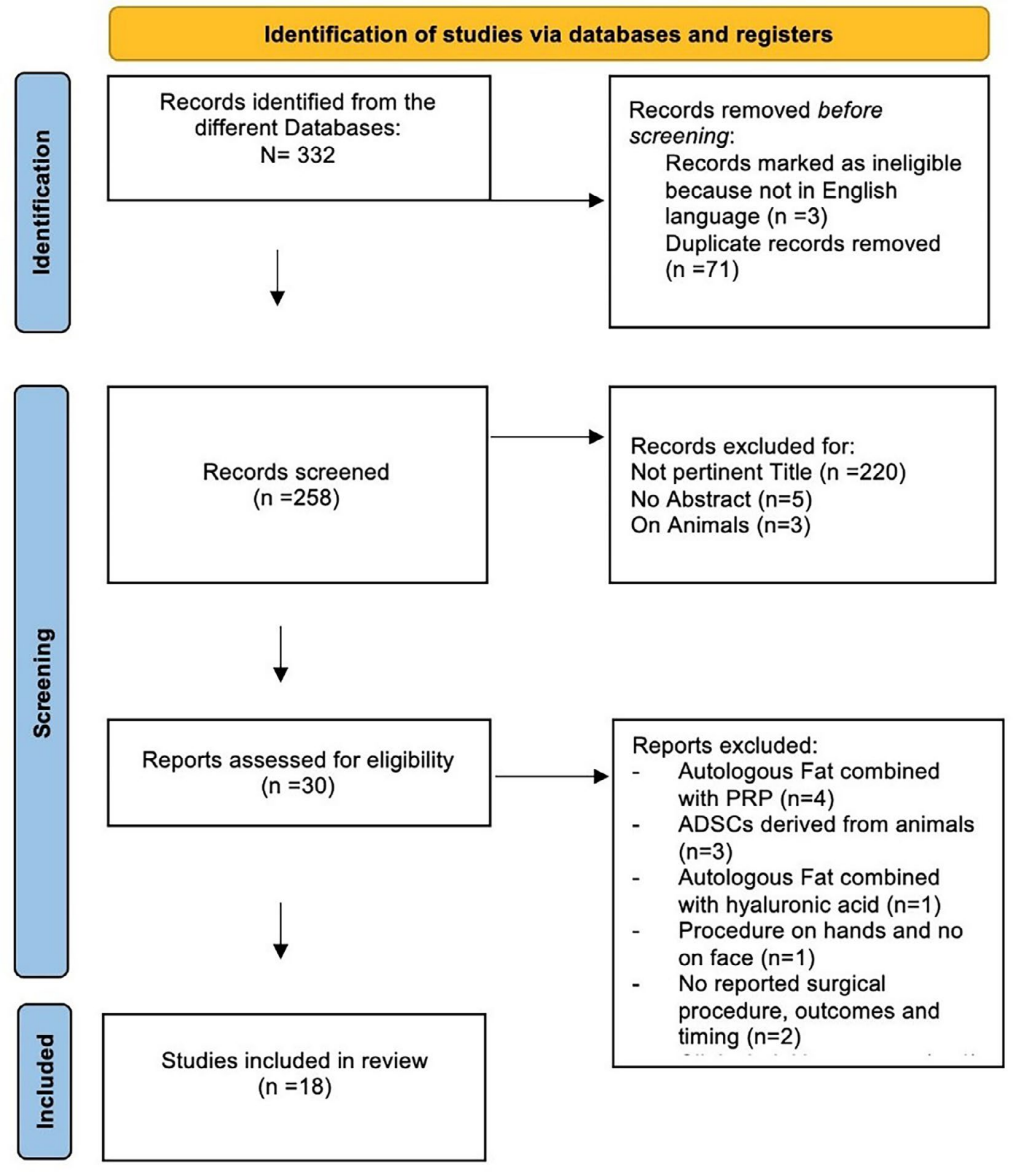
PRISMA 2020 flow diagram for new systematic reviews.

A total of 332 articles were initially identified from the different Databases; of these, three studies were excluded because they were not in the English language and 71 duplicated studies were removed. The remaining 258 papers were screened for the titles, so 220 not pertinent articles were excluded; thus, papers without abstracts (*n* = 5) and studies on animals (*n* = 3) were removed. Thirty articles were assessed for eligibility and, thus, scrutinised: after the exclusion of studies not satisfying the selection criteria, 18 studies were included in the systematic review [8, 9, 10, 11, 12, 13, 14, 15, 16, 17, 18, 19, 20, 21, 22, 23, 24, 25]. The PRISMA flow‐diagram is illustrated in Figure 1.

### 3.2 Studies Characteristics

The articles were divided into two tables, based on the main topic: the general features of the studies about the injection protocols were described in Table 1 while papers about the role of ASCs were shown in Table 2. In general, six studies were performed in Italy [7, 9, 14, 15, 21, 24], four in France [13, 16, 23, 25], three in the USA [12, 17, 18], two in the United Kingdom [11, 22], one in Israel [8], one in Slovenia [20] and one in Iran [10]. The design of most studies was observational prospective (55.6%); the remaining articles were narrative reviews (27.8%), case series (11.1%) and observational retrospective (5.5%). Not considering the narrative reviews, all the studies were performed in a single centre (Table 2).

**TABLE 1:** Features of the 12 included studies about fat grafting injection protocols.

Author/year	Study location	Study design	*N* cases (sex/age)	Surgical procedure	Timing of procedures	Outcomes	Complications
Arena 2022 [8]	Italy	Case series	5 3F 2M Age no reported	Autologous fat grafting (sedimentation): – macrograft (3.5 to 5 cc for side) for lip, perioral rim, and zygomatic area – micrograft (1.5 to 2.5 cc for side) nasolabial folds and the cheekbone area – nanograft (2 to 3 cc for side) for perioral wrinkles and vermilion border	1 procedure	At 6 months follow up improvement of: – elasticity of the skin – mouth opening	No complications
Berl 2022 [9]	Israel	Observational retrospective	17 14F 3M (range 27–72y)	Autologous nanofat graft (sedimentation) Total of 72 cc for full face: Forehead, temporal areas, checks, zygoma, upper and lower lips, nose, chin, mandibular areas (not specifying how many cc for each region)	1 procedure every 1 year	At 3 months follow up improvement of: – 0.85 cm in the absolute oral opening – Aesthetic appearance – Tissue elasticity and pliability	Complications spontaneously resolved in a few days: – Hematoma – Pain
Del Papa 2015 [10]	Italy	Observational prospective	20 20F (range 20–50y)	Autologous fat grafting (centrifugation 700xg for 3 min, only the intermediate layer was used). Total of 16 cc in the perioral region: – 6 cc upper lip – 6 cc lower lip – 4 cc opposite mouth corners	1 procedure	At 3 months follow up: – Increase of the interincisal distance (mean increase 2.63 mm) – Increase in the mouth perimeter (mean increase 9.2 mm) – Reduction of skin hardness evaluated with durometer – Increase of number of capillaries evaluated with capillaroscopy – Histological changes assessed by biopsies	Complications spontaneously resolved in about 2 weeks: – Ecchymosis
Gheisari 2018 [11]	Iran	Observational prospective	16 16F (range 29–54y)	Autologous fat grafting (sedimentation) 15–40 cc (average 27 cc) for Perioral region, upper lip, and lower lip, mouth corners, buccal, malar, and periorbital regions (not specifying how many cc for each region)	1 procedure	At 3 months follow up: – Mouth opening (0.78 cm) – Decrease of MHISS score – Aesthetic effect (fuller and softer face with less wrinkles) – Reduction of skin fibrosis (Rodnan skin score)	Complications spontaneously resolved in a few days: – Bruising
Griffin 2017 [12]	United Kingdom	Narrative review	90 Sex and age no reported	Autologous fat grafting (centrifugation 700gx3min, filtration PureGraft system, sedimentation) for a total of 8 cc in lips and perioral region: – 2 cc upper lip – 2 cc lower lip – 4 cc nasolabial folds	One procedure every 6 months	At 6 Months Follow‐Up, Improvement Of: – Mouth Opening (About 1 Cm Of Improvement) – Oral Sicca Syndrome – Aesthetic Aspect Of Facial Skin Fibrosis – Pain	No reported
Lagziel 2023 [13]	USA	Observational prospective	16 16F (range 20–65y)	Autologous fat grafting (sedimentation) for a total of 15 cc for lips and perioral region (not specifying how many cc for each region)	1 procedure in 1 year	At 1 year follow‐up, improvement of: – Mouth opening – Aesthetic aspect of facial skin fibrosis – Pain Approximately 50% fat resorption at the 1‐year mark.	No reported
Magalon 2015 [14]	France	Observational prospective	12 12F (range 20–65y)	Autologous microfat grafting (500 μm): (centrifugation 1200 g x 2 min; filtration PureGraft): – 6 cc upper lip – 5 cc lower lip – 4 cc nasolabial folds – 2 cc chin – 4 cc zygoma	1 procedure, in 1 year	At 1 year follow‐up, improvement of: – Mouth opening – Aesthetic aspect of facial skin fibrosis – Pain	Complications (rarely persisted more than 72 h): – Minimal bruising – Pain – Edema ecchymosis
Onesti 2015 [15]	Italy	Observational prospective	5 4F 1M (range 20–48y)	Autologous fat grafting (sedimentation) for a total of 16 cc for lips and perioral region: – 4 cc upper lip – 4 cc lower lip – 4 cc nasolabial folds – 4 cc marionette lines	Two procedures, 3 months apart, in 1year	At 1 year follow‐up, improvement of: – Mouth opening – Aesthetic aspect of facial skin fibrosis – Pain	No reported
Pignatti 2020 [16]	Italy	Observational prospective	25 19F 6M (range 25–65y)	Autologous fat grafting (sedimentation) for a total of 16 cc for lips and perioral region: – 4 cc upper lip – 4 cc lower lip – 4 cc nasolabial folds – 4 cc marionette lines	One procedure every 6 months (for a total of 3 in 1 year)	At 1 year follow‐up, improvement of: – Mouth opening (about 1 cm of improvement) – Oral sicca syndrome – Aesthetic aspect of facial skin fibrosis	Complications (rarely persisted more than 72 h): – Minimal bruising – pain – edema – ecchymosis
Sautereau 2016 [17]	France	Observational prospective	14 14F (range 50–60y)	Autologous microfat grafting (500 μm) (filtration): – 6 to 23 cc for all face (basing on patient's defect, not specifying how many cc for each region)	One procedure every 6 months (for a total of 3 in 1 year)	At 6 months follow‐up, improvement of: – Mouth opening (about 1 cm of improvement) – Oral sicca syndrome – Aesthetic aspect of facial skin fibrosis	Complications spontaneously resolved in a few days: – Minimal bruising – Pain – Perioral sensitive manifestation – Trigeminal neuralgia
Strong 2019 [18]	USA	Narrative review	83 Sex no reported (range 17–41y)	Autologous fat grafting (centrifugation, filtration and sedimentation): – 10 cc forehead, scalp and glabellar region – 6 cc lips, perioral region and chin.	Two procedures, 3 months apart, in 1 year	Features of scleroderma such as inflammatory hyperpigmentation, hypopigmentation, or visible vessels, not improve with fat graft; At 1 year follow‐up: – On the forehead 51% to 75% of improvement – On the chin less than 25% of improvement – On the lips and perioral region 65% to 79% of improvement	Complications (rarely persisted more than 72 h): – minimal bruising – Pain – Edema – Erythema
Strong 2021 [19]	USA	Observational prospective	10 10F (range 25–78y)	Autologous fat grafting (centrifugation 3000 rpm x 3 min and sedimentation): – 10 cc forehead, scalp and glabellar region – 8 cc lips – 6 cc nasolabial folds – 17 cc malar region – 8 cc marionette lines – 3 cc chin	Two procedures, 6 months apart, in 1 year	At 6 months follow‐up, improvement of: – Mouth opening – Oral sicca syndrome – Aesthetic aspect of facial skin fibrosis – pain	Complications spontaneously resolved in a Few days: Minimal bruising – Pain – Minimal bruising – swelling

**TABLE 2:** Features of the six included studies about the adipose tissue‐derived stem cells role.

Author/year	Study Location	Study Design	N cases (sex/age)	Objective/procedure	Outcomes
Brezovec 2021 [20]	Slovenia	Narrative review	No reported	Focus on local effects of adipocytes in the adipose to mesenchymal Transdifferentiation (AMT), effects of the adipose stromal vascular fraction on SSc pathogenesis and Systemic effects of adipose tissue secretome.	– Lipolysis contributes to fibrogenesis through AMT differentiation and release of fatty acids (FA) – Unbalanced metabolism of FA can play a role in SSc development – Other adipose tissue secretome molecules (e.g., lysophosphatidic acid), novel adipokines and extracellular vesicles from adipose mesenchymal stem cells make important contributions to the pro‐/antifibrotic balance – Adipocyte derived myofibroblasts may represent a unique transcriptomic population
Del Papa 2020 [21]	Italy	Observational prospective	10 F	Comparation between ASCs in SSc patients and in normal patients in normoxic and hypoxic conditions	– Normoxic and hypoxic conditions do not modify the proliferation rate of both normal and SSc ASCs – Hypoxia significantly increased mRNA expression of VEGF by HD and SSc ASCs but had no effect on the mRNA expression of pro‐fibrotic mediators – Normal and SSc fibroblast proliferation was significantly reduced in both co‐culture systems (*p* < 0.001) – Protein secretion of TGF‐β1, CTGF and Col I were significantly reduced (*p* = 0.003, *p* = 0.02, *p* = 0.04) – mRNA expression and production of VEGF was observed in SSc fibroblasts cultured in the presence of normoxic and hypoxic CM (*p* = 0.002 and *p* < 0.0001, respectively).
Griffin 2017 [22]	United Kingdom	Case series	6 F (range 28–40 y)	Comparation between ASCs in SSc patients and in normal patients	– No alteration in the phenotype or surface antigenexpression of ASC‐SSc compared to ASC‐N – Differentiation capacity equivalent – ASC‐SSc did not display any morphological or adhesive abnormalities – The proliferation rate and metabolic activity of ASC‐SSc was reduced (*p* < 0.01) – The migration and invasion capacity of ASC‐SSc was reduced (*p* < 0.01)
Maria 2017 [23]	France	Narrative review	No reported	Focus on the role of ASCs in SSc	– MSCs/ASCs display immunosuppressive, antifibrotic, pro‐angiogenic, and anti‐oxidative responses – Reduction of skin is characterised by tissue downregulation of collagen 1/3, α‐SMA, and TGFβ1 expression at the mRNA level, total collagen deposition in tissue, inhibition of SMAD2/3 pathway and histological evidence.
Rosa 2021 [24]	Italy	Narrative review	No reported	Focus on the double role of ASCs in SSc: therapeutic in local treatment and pathological in systemic administration	– ASCs present a variety of advantages, including ease and non‐invasiveness of collection, as well as higher differentiation ability and regenerative properties – The disease microenvironment may substantially affect the fate of ASCs by directing their pathologic activation (pro‐fibrotic action) – ASC‐based therapeutic application in SSc has been shown to be effective only for the local treatment of facial and hand cutaneous manifestations, while very little is known about their possible efficacy at the systemic level.
Velier 2019 [25]	France	Observational prospective	7 F Age no rep.	Phenotype, senescence, differentiation potential, and molecular profile were determined in ASCs in SSc patients and in normal patients.	– Differentiation capacity, senescence, and mRNA – Profiles did not differ significantly between ASC‐SSc and ASC‐N – ASC‐SSc retained the ability to stimulate angiogenesis through paracrine mechanisms; however, functional assays revealed reduced potential compared to ASC‐N. – DF fibrosis markers were significantly decreased after co‐culture with ASC‐SSc. – SSc effects do not significantly compromise angiogenic and antifibrotic paracrine properties of ASC

### 3.3 Data Analysis

#### 3.3.1 General Features of Articles About Injection protocols [8, 9, 10, 11, 12, 13, 14, 15, 16, 17, 18, 19]

A total of 313 patients with facial manifestations of systemic scleroderma were considered in this systematic review; 91.4% of cases were females, with a F:M ratio of 10.7:1. The average age was 42.5 (range: 17–78 years). The most commonly reported facial symptoms were perioral rhytids, skin tightening and hardening, accentuated nasolabial and marionette wrinkles, malar volume depletion, mouth opening limitation, pain, oral sicca syndrome and quality of life impairment.

#### 3.3.2 Fat Grafting Harvest, Processing Mode and Injection [8, 9, 10, 11, 12, 13, 14, 15, 16, 17, 18, 19]

In all cases autologous fat graft was harvested: in 70% of cases fat was processed by sedimentation; in the remaining 30% of cases, centrifugation and filtration were used to process adipose tissue. In particular, for the centrifugation mode, different settings were reported in revolutions per minute (rpm), whereas other settings were reported as *g*‐force (g); 1200 rpm × 2 min was used to harvest microfat, while 1300 rpm for 10 min was used for nanofat.

The total volume of injected fat graft and the number of treatments were different between patients, considering the volume depletion and the skin elasticity at the time of presentation. In general, the volume of injected fat ranged from 6 cc for only the perioral region to 72 cc for a full‐face approach; in particular, the specific volumes injected in the different regions were:

- 2–6 cc for the lower lip;
- 2–6 cc for the upper lip;
- 4–8 cc for the nasolabial folds;
- 4–8 cc for the marionette lines;
- 2–6 cc for the chin;
- 4–17 cc for the malar region;
- 10 cc for the forehead and the glabellar region;
- 10 cc for the scalp.

The timing of the procedures varied greatly, ranging from one procedure in 1 year to one procedure every 3 months. In particular, in 50% of cases, one procedure in1 year was reported; in 33.3%, one procedure every 6 months was described, while in the remaining 16.7% of cases, two procedures were repeated 3 months apart in 1 year.

#### 3.3.3 Outcomes and Complications [8, 9, 10, 11, 12, 13, 14, 15, 16, 17, 18, 19]

Regarding the follow‐up, in 41.7% the clinical outcomes were evaluated at 1 year, in 33.3% at 6 months while in the remaining 25% performed at 3 months follow‐up. In all cases, the following improvements were recorded:

- Increase in mouth opening from 0.85 to 1 cm;
- Reduction of skin hardness evaluated with durometer;
- Increase in the number of capillaries evaluated with capillaroscopy;
- Xerostomy reduction;
- Decrease of facial skin fibrosis evaluated with Rodnan skin score;
- Decrease of pain.

Regarding the complications, the most reported complications were a minimal bruising (50% of studies) and pain (50% of studies); other recorded complications were swelling and ecchymosis (41.7% of studies), perioral sensitive manifestation and trigeminal neuralgia (8.3% of studies).

#### 3.3.4 Role of ASCs in Improvements of Facial Skin Elasticity and function [20, 21, 22, 23, 24, 25]

In the articles about the role of ASCs, 23 women with SSc underwent sampling of adipose tissue to study the characteristics of these cells. The underlying mechanism of action of ASCs was suppressing excessive collagen synthesis and expediting the collagen remodelling process through the secretion of cytokines that inhibit fibroblast differentiation into myofibroblasts and the production of collagenase [20, 21, 22]. In inflammatory microenvironments, such as in scleroderma, the immunomodulatory effects of ASCs contributed to enhancing tissue structure by inhibiting local inflammation. This capability enabled them to effectively disrupt the inflammatory response, offering potential benefits for treating individuals with scleroderma. Adipose stem cells resulted in secreting factors like indoleamine 2,3‐dioxygenase and nitric oxide, which respectively induced apoptosis in T cells and natural killer cells responsible for the abnormal deposition of collagen, proteoglycans and elastic fibres [21]. Additionally, these stem cells demonstrated the secretion of anti‐inflammatory cytokines, including interleukin‐10, which inhibited the release of pro‐inflammatory mediators, reduced antigen presentation and diminished phagocytosis [24, 25]. Collectively, these findings suggested that one of the mechanisms through which fat grafting enhanced outcomes in scleroderma patients was by limiting inflammation. Fat grafting demonstrated, moreover, the ability to enhance angiogenesis by serving as a rich source of cells and growth factors that facilitated angiogenesis and supported the survival of endothelial cells [23]. Both endothelial cells and vascular smooth muscle cells have been observed to contribute to the formation of new blood vessels, supplying nutrients to the newly transplanted fat. Additionally, adipose stem cells played a crucial role by providing growth factors to the vascular cells in a paracrine manner. Collectively, these investigations suggested that fat grafting had the potential to stimulate angiogenesis by providing the necessary cell types for the generation of new blood vessels [24, 25].

In conclusion, the review of these articles confirmed that fat grafting diminished the clinical manifestations of fibrosis, likely attributed to the effects of the stem cells present in the processed adipose tissue. Adipose stem cells released a profusion of anti‐inflammatory cytokines that mitigate the transformation of fibroblasts into myofibroblasts, consequently reducing the deposition of collagen and other extracellular matrix proteins in the skin and organs. Moreover, adipose stem cells, when exposed to stressful environments, secreted an abundance of matrix metalloproteinases and collagenase capable of degrading the extracellular matrix. These findings strongly suggested that the stem cells component of the fat graft had the potential not only to prevent the deposition of collagen and other extracellular matrix proteins but also to reverse the underlying fibrosis.

## 4 Discussion

The facial manifestations of SSc are very frequent, occurring in about 90% of cases of the diffuse forms of this pathology. The mechanism underlying these manifestations is the excessive fibrosis and vascular deregulation tissue damage, mediated by vascular endothelial growth factor (VEGF in particular isoform A involved in angiogenesis process), endothelin 1, platelet‐derived growth factor (PDGF), in particular the isoform BB that is related to inflammation, basic‐fibroblast growth factor (FGF‐b), overexpression of collagen and tumour necrosis factor alpha (TNF‐α) and chemokine alterations. The skin fibrosis determines an a‐mimic face, a development of vertical furrows around the mouth, a sharp nose, thin lips, occurrence of telangiectasias and pigmentation abnormalities (hypopigmentation and hyperpigmentation); the salivary gland and mucosal fibrosis determines Oral Sicca syndrome (in approximately 70% of patients with SSc), decrease of mouth opening (of about 40%) and pain during chewing and swallowing movements, with consequent limitation of feeding and dental care [17].

Over the years, the conventional treatment was based on a facial stretching programme approach, such as connective tissue massage, kinesitherapy and Kabat's technique [26], or systemic therapies, such as Endothelin receptor antagonists, Rituximab, recombinant human erythropoietin, granulocyte colony stimulating factor (G‐CSF) and calcium channel blockers [27], phototherapy and intense pulsed‐light treatment [28]. However, these solutions on one side depend on the continuity of the treatment and on the availability of materials/equipment; on the other side, they can expose the patient to adverse/allergic reactions and they do not have a regenerative effect [29]. Thus, in recent years, autologous fat transplantation as a regenerative and translational approach [30] has become a valid alternative to conventional treatment, due to easy accessibility and absence of foreign material reactions.

The effectiveness of adipose tissue in the treatment of SSc has been already demonstrated in literature by different authors. The mechanism underlying the effect of fat graft is supported by ASCs that may reverse the fibrotic pathway, modulating the angiogenesis, the immune response and the transforming growth factor beta‐1 (TGFβ1) signal [5, 12]. In particular, recent scientific research has shown that fat grafting is able: ‐ to improve angiogenesis by providing growth factors to promote endothelial cell survival; −to reduce fibrosis by increasing collagenase activity and limiting deposition of extracellular matrix proteins; −to provide stem cells that, by proliferating and differentiating, can be a tissue support [22, 23, 25, 31, 32]. In particular, both basic research and clinical observations have indicated that fat grafting exerts a therapeutic impact on scleroderma, enhancing skin fibrosis and diminishing pigmentation [12, 33].

According to the literature, there are multiple donor sites for fat harvesting. In particular, the lower abdomen and inner thigh contain a higher density of ASCs. Those of the thigh have a higher angiogenic and adipogenic potential [34].

Some authors affirm that fat harvesting by barbed cannula is superior to the smooth one because it gives greater availability of ASCs. Moreover, the harvesting should be performed in the superficial layer of the subcutaneous tissue because it contains more stromal vascular fractions (SVFs) cells [35, 36, 37].

After the harvesting, the fat graft must be distributed diffusely, with very moderate injections and through small cannulas. Post‐procedure, the injected region must remain immobile for approximately 5–7 days to support the function of the ASCs and prevent damage to the new vessels [35].

However, the concepts that are still a challenge for many authors concern the appropriate timing of administration and the right amount of fat to inject, in order to obtain more effective and lasting results. Wang et al. [6], in their article, confirm the confusion about the current clinical practise, questioning how and when to perform the procedure. Strong et al. [18], in their study, had laid the foundations for treatment guidelines for SSc with autologous fat grafting; however, confirming the need for further studies to investigate the timing and the number of treatments, the volume and the site of injection, the effect on the recipient site and the longevity of the fat grafting.

Therefore, the authors of this paper carried out a systematic review of all the available literature on this topic up to August 2023, to provide a therapeutic protocol regarding the methods and timing of the fat graft procedure, based on the biological features of ASCs.

First of all, it is paramount to determine the optimal time to begin treatment: several authors recommend performing the first procedure at earlier stages of the disease, before limited facial expression, preventing the irreversible deformity that can result from the skin fibrosis and hardening. Moreover, Strong et al. [18] advise starting fat administration earlier than vascular compromise and in a passive phase of the disease. In fact, the authors have hypothesised that when the lesions are active, as when there are signs of spreading, the chance of fat survival decreases [18, 38]. Thus, the analysis of the studies included in the systematic review confirms that it is advisable to start the treatment before the onset of these complications.

Regarding the processing procedure of fat, the three main reported techniques are centrifugation, gravity sedimentation (decantation) and filtration [39]; to date, there is no unanimous consensus regarding specific procedural guidelines and indications, but the analysis of our data reveals that for most authors, filtration more effectively eliminates contamination from other cells and has a higher long‐term retention rate [17, 18, 40]. For other authors, centrifugation at 700/1200 g for 3 min preserves intact ASCs with improved cellular functions and increases SVF density in proportion to the depth of harvest [10, 12, 14, 19, 35]. Despite this evidence, challenges persist in achieving substantial fat retention. Emerging techniques in fat grafting may offer potential solutions to address this intricate issue, but further research is needed to substantiate their efficacy. For example, Nanofat grafting is extracted using a mechanical process that disrupts most mature adipocytes and certainly does not achieve true nanoscale standards [41]. Nanofat grafting is deemed suitable for tissue revitalization, such as improving skin texture, and it can enhance the concentration of cytoactive ASCs. Otherwise, SVF is obtained from mechanicallydigested fat tissue and encompasses a heterogeneous cell population, including endothelial cells, haematopoietic cells and a minor fraction of ASCs (less than 3%) [42]. However, there is still no evidence in the scientific literature that these alternative methods of adipose tissue purification are effective in treating patients with facial scleroderma. In vitro studies are promising, as these mechanical methods can isolate the SVF or ASCs, making them potentially more effective for targeting scleroderma [43, 44, 45].

**FIGURE 2:**
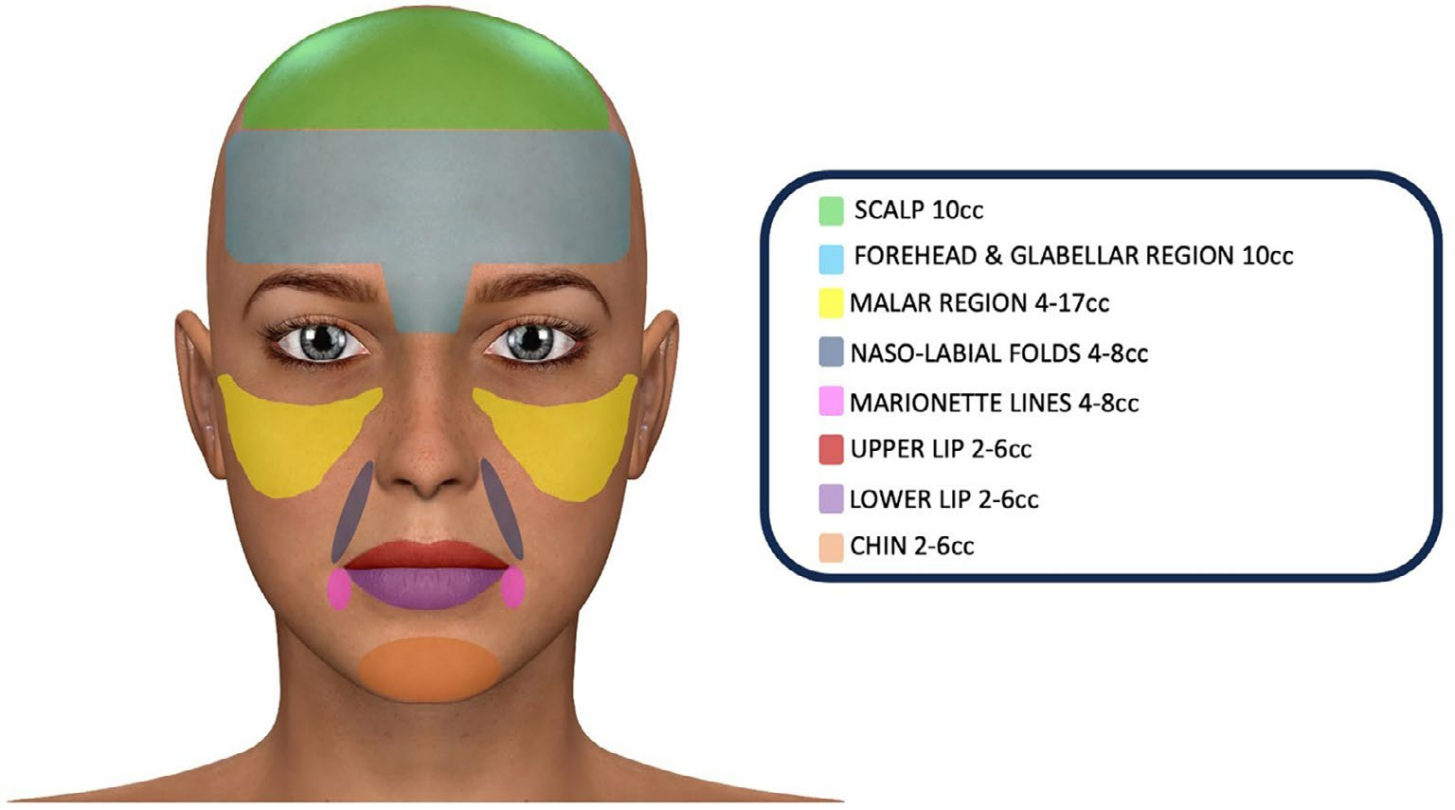
New injection protocol of fat grafting for facial manifestations of scleroderma.

The second key point is to establish how much fat to inject into the various facial regions and at what time interval: several authors have hypothesised that a second round of fat grafting gives better and longer‐lasting results because with the first injection the skin has improved its elasticity and laxity and is ready to receive larger volumes of fat [46, 47]. Therefore, many authors recommend performing two or additional rounds of fat transplant spaced out by months according to the severity of the pathology [19, 23, 25]; our systematic review reveals that it is advisable to perform one injection at time 0, then another infiltration after 3 or 6 months, according to the severity of the clinical manifestations, due to the higher skin elasticity and laxity after the first procedure, and finally maintenance infiltrations every 6 months for the first 2 years and then once a year. In fact, a general fat resorption of about 50% was recorded in all the analysed studies at 1 year follow‐up; in particular, Strong et al. [18] reported a different resorption according to the considered regions: 25%–49% in the forehead and glabellar region, 75% in the chin and 21%–35% in the lips and perioral region, probably related to the applied muscle force. Consequently, the need to inject the right minimum quantities of fat into specific facial regions appears to be mandatory, in order to obtain less reabsorption and lasting results. For this reason, by processing the data of our systematic review, we have proposed a basic injection protocol to consider for the first fat transplant, adjusting the quantities within the indicated ranges, based on the initial skin elasticity and on the disease severity (Figure 2). This protocol can be useful on the one hand as a treatment guideline aimed at obtaining minimal fat resorption, on the other hand to avoid injecting excessive amounts of fat that could stretch or even ischemize the skin. It is advisable to avoid an over‐correction greater than 20%, both because the greater tension can cause ischemic skin necrosis and because the central core of the fat graft may not be sufficiently vascularized. In this case, the fat graft reabsorbed more quickly [6].

As reported in the literature, the addition of stem cells and platelet concentrates such as platelet rich plasma (PRP), platelet rich fibrin (PRF) and concentrated growth factor (CGF) to fat has been proposed to prolong its retention at the receiving site and to increase its quality. In addition, PRP has an antiphlogistic action, promotes coagulation, stimulates tissue regeneration and in combination with fat has a synergistic effect in patients with SSc [35, 48, 49]. Other techniques to redefine the face have been described [50], although their use has not been specific to scleroderma patients. An interesting element that emerges from the review is that the disease microenvironment may substantially affect the fate of ASCs by directing their pathologic activation (pro‐fibrotic action): in fact, in local administrations (face, hands) an anti‐inflammatory and regenerative mechanism is activated while in systemic administrations (organs) a pro‐fibrotic mechanism could be activated [24]. For this reason, fat grafting is currently recommended only for the treatment of facial and hands manifestations, but not for systemic treatments.

Our future perspective will be to carry out an observational prospective study, employing our proposed injection protocol.

The limit of our study is that we were unable to perform a meta‐analysis due to the mainly qualitative and non‐quantitative data results.

## 5 Conclusions

The facial manifestations of SSc can be very disabling for the patient both in aesthetic and functional terms. Many authors have already demonstrated that autologous fat grafting can be a valid treatment to improve signs and symptoms of this disorder, but a standard protocol on the method and timing of administration has not yet been defined. Our study aims to define a standardised protocol, performing a systematic review on the topic. The data obtained from our systematic review seem to confirm that autologous fat grafting is a valid treatment for the improvement of the facial manifestations of SSc, due to the ASCs' properties of suppressing excessive collagen synthesis and inhibiting local inflammation. The clinical relevance of this study consists of providing a standardised protocol that can guide the surgeon to obtain a lower resorption rate and more long‐lasting improvements in terms of aesthetic and functional outcomes. In particular, it seems to be appropriate to carry out two administrations at 3 or 6 months apart, according to the severity of the case, and then an annual maintenance administration. It is important to inject pre‐established minimum quantities of fat to the different facial regions to obtain longer‐lasting results and to avoid excessive resorption. For this reason, we believe that following our injection protocol based on the systematic review data, the resorption rate can be lower and the improvements can be more lasting. On the other hand, the administration for the treatment of systemic symptoms is not recommended.

## Author Contributions

Antonio Arena: conceptualization; Stefania Troise: methodology and writing – review and editing; Francesco De Francesco: writing – review and editing; Michela Apolito: writing – original draft preparation; Umberto Committeri: writing – original draft preparation; Giovanni Salzano: investigation and resources; Antonio Romano: investigation and resources; Paola Bonavolontà: investigation and resources; Vincenzo Abbate: data curation; Riccardo Nocini: data curation; Giovanni Dell'Aversana Orabona: project administration and final approval.

## Consent

The authors have nothing to report.

## Conflicts of Interest

The authors declare no conflicts of interest.

## Data Availability

All the data are available in the present manuscript.
